# Musculoskeletal Ultrasound Reliability in Knee Osteoarthritis: A Pilot Study of Cartilage Thickness, Osteophytes and Weight-Bearing Meniscal Extrusion

**DOI:** 10.3390/medicina62071292

**Published:** 2026-07-04

**Authors:** Mihaela Minea, Andreea-Alexandra Lupu, Ghiulcin Nurla, Alexandra-Elena Minea, Liliana Vlădăreanu, Viorela-Mihaela Ciortea, Laszlo Irsay, Mădălina-Gabriela Iliescu

**Affiliations:** 1 Faculty of Medicine, Doctoral School, “Ovidius” University of Constanta, 1 University Alley, Campus-Corp B, 900470 Constanța, Romania; mihaeala.minea@365.univ-ovidius.ro; 2 Hospital Rehabilitation Unit, Balneal Sanatorium of Techirghiol, Victor Climescu Street 34–40, 906100 Constanța, Romania; nurlaghiulcin@gmail.com (G.N.); liliana.vladareanu@365.univ-ovidius.ro (L.V.); 3 Department of Medical Rehabilitation, Faculty of Medicine, “Ovidius” University of Constanța, 1 University Alley, Campus-Corp B, 900470 Constanța, Romania; 4 Faculty of Medicine, “Carol Davila” University of Medicine and Pharmacy, 37 Dionisie Lupu Street, 020021 Bucharest, Romania; alexandra-elena.minea0720@stud.umfcd.ro; 5 Department of Rehabilitation Medicine, University of Medicine and Pharmacy “ Iuliu Hațieganu”, Victor Babeș Street 8, 400012 Cluj-Napoca, Romania; viorela.ciortea@umfcluj.ro (V.-M.C.); laszlo.irsay@umfcluj.ro (L.I.)

**Keywords:** knee osteoarthritis, musculoskeletal ultrasound, meniscal extrusion, cartilage thickening, weight-bearing, reproducibility

## Abstract

*Background and Objectives:* Musculoskeletal ultrasound (MSUS) is increasingly used to assess structural changes in knee osteoarthritis (KOA), although measurement reproducibility may vary with examination protocol and operator experience. This pilot study aimed to evaluate intra- and inter-observer reliability for assessing cartilage thickness, osteophyte dimensions and meniscal displacement in supine and weight-bearing conditions in patients with KOA. *Material and Methods:* This prospective reliability study included patients with KOA stages 2–3 on the Kellgren–Lawrence (K-L) scale. Ultrasound (US) evaluation of cartilage thickness and osteophytes was performed in the supine position, while medial and lateral meniscal extrusion was additionally assessed in bipedal and single-leg conditions using a standardized protocol. Intra- and inter-reproducibility were evaluated through the intraclass correlation coefficient (ICC), agreement analysis and kappa statistics. The analysis was performed using Microsoft Excel (version 2019) and IBM SPSS version 26 software. *Results:* Both inter-reliability and intra-reliability were evaluated, with intra-rater agreement being constantly higher than inter-rater agreement. From the inter-rater reliability, ICC values ranged from 0.48 to 0.88 for quantitative ultrasound measurements, whereas osteophyte assessment showed Cohen’s k values ranging from 0.71 to 0.86, indicating substantial to almost perfect agreement. Both evaluators identified a higher number of knees with medial meniscal extrusion > 3 mm under weight-bearing conditions than in the supine position. *Conclusions:* Standardized US evaluation demonstrated at least good reproducibility for structural abnormalities in KOA. Medial and lateral meniscal displacement increased under loading conditions, highlighting the added value of dynamic weight-bearing assessment. However, the clinical significance of load-dependent meniscal extrusion requires further investigation.

## 1. Introduction

Knee osteoarthritis (KOA) is whole-joint disease, characterized by complex interactions between cartilage degeneration, meniscal pathology, osteophyte formation, subchondral bone remodeling and synovial inflammation [1]. These structural changes alter joint biomechanics, contribute to pain and functional impairment, and play a role in disease progression. Recent evidence indicates that osteoarthritis is a complex disorder involving structural, biomechanical, inflammatory and metabolic mechanisms that collectively contribute to disease progression [2]. In addition, metabolic factors, including alterations in element homeostasis, have recently been recognized as potential contributors to cartilage degeneration and osteoarthritis progression [3].

In clinical practice, there is frequent discordance between symptom severity and radiographic findings. Minimal structural changes are often reported in patients with significant symptoms, and important radiographic lesions may be associated with only mild clinical features. Once considered a degenerative condition, it is now understood as a process involving cartilage degradation, subchondral bone remodeling, meniscus degeneration and inflammatory changes in the synovium. An appropriate assessment of these structural changes is important for understanding symptom variability and disease progression. Radiography (X-ray) examinations provide limited information, so detailed imaging methods are needed [4].

In recent decades, musculoskeletal ultrasound (MSUS) has emerged as a practical and accessible tool for rheumatological musculoskeletal disorders [5]. Previous research has shown that the method is valuable not only for inflammatory joint diseases but also for degenerative conditions, providing a detailed assessment of articular and periarticular structures. Furthermore, ultrasound (US) has increasingly been recognized as a useful tool in osteoarthritis (OA), enabling visualization of structural changes such as detection of osteophytes, meniscus displacement, cartilage focal damage and joint inflammation, in the early stage that may not be fully captured by conventional X-ray [6,7]. Regarding cartilage thickness, previous US studies reported normal values ranging from 1.8 mm to 2.5 mm, depending on sex and measurement site [8]. Meniscal extrusion is an important structural abnormality associated with structural degeneration, root tears, changes and disease progression. Although different US thresholds have been proposed, medial meniscal extrusion greater than 3 mm is the most widely accepted criterion for pathological extrusion [9,10]. No clear consent exists for pathological lateral meniscus extrusion. Furthermore, dynamic evaluation of meniscal protrusion under different loading conditions, following established protocols, may provide additional clinically relevant information [11,12,13].

In parallel with imaging-based approaches, several emerging technologies have been proposed for the functional assessment of knee osteoarthritis. Vibroarthrography (VAG) and vibroacoustic signal analysis evaluate acoustic and vibrational signals generated during joint motion and have shown promising results for non-invasive assessment of joint function and biomechanical alteration associated with osteoarthritis. These non-invasive techniques may provide complementary functional information alongside conventional imaging methods. Nevertheless, unlike these signal-based approaches, musculoskeletal ultrasound allows direct visualization and quantitative assessment of anatomical structures while also enabling dynamic evaluation under physiological loading conditions, thereby combining structural and functional information within a single examination [14,15].

Standardized definitions of pathology and assessment protocols for each anatomical area were developed through initiatives such as Outcome Measures in Rheumatology (OMERACT) [16,17]. As a result, US reproducibility across operators in the centers improved [18,19]. In a recent study, Menyawi et al. reported a correlation with clinical and radiographic changes and improved inter-evaluator agreement, including that of junior examiners, when a predefined cut-off value was applied, thereby demonstrating the validity, reliability and feasibility of the OMERACT ultrasound score for KOA patients [20].

Given the operator-dependent nature of MSUS and potential variability in equipment and acquisition techniques, local method validation across different clinical environments is also important to confirm the consistency of these findings for diagnosis, therapeutic selection and research [11].

### Objective of the Study

The aim of this study was to evaluate intra- and inter-observer reliability of a standardized MSUS protocol for assessing cartilage thickness, osteophyte presence and size, and the degree of meniscal extrusion across different conditions (supine, standing and single-leg bearing) in patients with KOA admitted to the Balneal and Rehabilitation Sanatorium of Techirghiol (B.R.S.T.), prior to its implementation in larger clinical studies.

## 2. Materials and Methods

### 2.1. Study Design and Participants

A cross-sectional study was conducted over a six-week period at the B.R.S.T., including 25 patients admitted for rehabilitation, with bilateral KOA, grades 2–3 K-L (a total of 50 knees). Approval from the B.R.S.T. Ethical Committee was obtained before the study’s initiation (no. 26/27 November 2024). All participants were informed about the clinical evaluation, X-ray examination and US assessment procedures. Written consent was obtained from each patient before enrolment in this study.

#### Inclusion and Exclusion Criteria

Subjects aged 40 years or older with bilateral KOA, diagnosed according to the American College of Rheumatology (ACR) clinical criteria and graded 2–3 on the K-L classification, were enrolled in this study. Restricting the cohort to K–L grades 2–3 ensured measurable structural changes while reducing bias from very advanced disease stages, which may affect reliability estimates.

Patients were excluded if they had secondary causes of KOA, including inflammatory autoimmune rheumatic diseases, crystal-induced arthropathies or infectious arthritis. Additional exclusion criteria were a history of lower-limb surgery, significant trauma, congenital disorders or associated neurological conditions, given their potential effects on lower-limb biomechanics and the secondary structural changes that could influence ultrasound measurements. Patients unable to maintain the single-leg weight-bearing position with minimal support were also excluded, as they did not meet the evaluation protocol requirements.

### 2.2. Assessments

#### 2.2.1. Clinical and History Data Assessment

For all patients, demographic and clinical history data were collected using a study-specific questionnaire. The following variables were recorded: age, height and weight, body mass index (BMI), pain level assessed using the Numeric Rating Scale (NRS), a simple and widely used tool for pain evaluation in patients with KOA, and time since diagnosis.

#### 2.2.2. Radiological Assessment

All participants had undergone a standardized radiographic evaluation of the knee joint before inclusion and disease severity had been graded using the K-L classification.

#### 2.2.3. Musculoskeletal Ultrasound Protocol Assessment

The US protocol, including patient positioning, acquisition conditions and evaluator-related variables, was designed in accordance with current European League Against the Rheumatism (EULAR) recommendations for MSUS studies [21]. The knee joint evaluation was performed using an Essaote MyLab X6 System (Esaote S.p.A., Genoa, Italy) with a 4–15 MHz linear transducer (SL1543, Esaote S.p.A., Genoa, Italy). Machine settings, including depth, gain and focus, were adjusted according to the anatomical structures under examination and the patient’s characteristics to optimize image quality. The examinations were performed by two independent evaluators with prior MSUS experience of >15 years and >10 years, respectively. Each patient was assessed twice, with a 48 h interval between examinations, at the same time of day (between 10 and 12 a.m.). The 48 h interval was selected to minimize recall bias while ensuring stability of structural findings, with minimal risk of relevant change in joint effusion over this period. Cold and hot applications or exercise therapy were not used within 1 h before the examination.

For the intra-rater reliability analysis, the primary observer repeated all ultrasound measurements after the predefined interval without access to the previous measurements. For the inter-rater reliability analysis, each observer independently performed and recorded the measurements and was blinded to the other observer’s results.

The quality of the captured images was semi-quantitatively assessed by each examiner, with scores ranging from 1 to 3 as follows: 1 (good = meniscus, cartilage, osteophytes fully visible), 2 (medium = subchondral or cortical line partially outlined) or 3 (low = shadows, low echo, difficult to measure). The time required by the evaluator for each exam was also recorded at the end of the assessments.

Before this study began, the two examiners developed a standardized ultrasound assessment protocol in line with the EULAR guidelines [22,23]. The US assessment was conducted systematically, dynamically, multiplanar and bilaterally, primarily using grayscale imaging, with Doppler applied when necessary. To ensure the clarity of the examination, structures were checked in both longitudinal and transverse planes.

Longitudinal and transverse scans of the femoral cartilage were performed with the patient in the supine position and the knee maximally flexed to optimize visualization. The probe was placed transversely in the suprapatellar region, perpendicular to the femoral cartilage surface, to obtain an axial view of the femoral trochleae. Cartilage thickness was measured at three standard locations: the intercondylar region, the medial femoral condyle and the lateral femoral condyle. Cartilage thickness was evaluated as the distance between the cartilage–synovial space interface and the subchondral bone interface [23,24,25]. The standardized ultrasound examination protocol, including patient positioning, transducer placement and measurement techniques, is illustrated in Figure S3 as Supplementary Material.

A longitudinal US scan of the medial and lateral tibiofemoral joints was performed with the patient in the supine position and the knee flexed to approximately 15° [22]. Bony step-up prominences arising from the cortical margin of the bone contour or the articular margins, with or without posterior acoustic shadowing, were identified in two perpendicular planes and classified as osteophytes. For objective assessment, osteophyte size was measured as the maximum length in the longitudinal plane (mm) [17]. The semi-quantitative grading system, based on OMERACT recommendations (0–3), was used, with preset grading: 0 indicating absence, 1 (<2 mm) indicating small osteophytes, 2 (2–4 mm) indicating moderate osteophytes and 3 (>4 mm) indicating large osteophytes that clearly deform the joint margin. The size thresholds were used to categorize severity [11]. This approach aimed to ensure the consistent inclusion of all osteophyte sites in the statistical analysis, thereby reducing operator dependence and improving inter-examiner consistency.

In the same patient position, the peripheral portions of both menisci were evaluated. Meniscal displacement was assessed in accordance with OMERACT recommendations. The line between the tibial and femoral cortices was defined, and the distance between it and the most peripheral margin of the meniscus was measured and reported in mm [26,27]. Meniscal assessment was further conducted in the standing position and under one-leg weight-bearing conditions to evaluate the impact of additional mechanical load on the knee joint during upright stance and ambulation. The participants were positioned upright, with their knees approximately 15 cm apart to allow adequate transducer placement to evaluate internal compartments. During the single-leg weight-bearing assessment, participants were allowed to lightly support themselves using a fixed object solely for balance without unloading weight from the examined limb. Meniscal displacement was assessed in both the medial and lateral compartments in both standing positions and reported in millimeters (mm), with differences observed across positions. Given that a medial meniscal extrusion threshold greater than 3 mm is widely considered pathological in the literature, the number of knees exceeding this limit was recorded in each of the three evaluated positions [12].

The evaluation protocol was limited to assessing cartilage thickness, identifying and measuring osteophytes and evaluating meniscal displacement across the three postures. Effusion (defined as a maximum anteroposterior diameter of the suprapatellar recess > 4 mm) and synovial hypertrophy (the presence of abnormal hypoechoic, non-displaceable and poorly compressible tissue that may show a vascular signal) were not systematically assessed and were therefore excluded from the statistical analysis [17].

### 2.3. Data Collection

For the assessed and measured anatomical structures, each examiner recorded the values for both evaluations in Microsoft Excel. The internal and external meniscal extrusion in supine, bipedal and single-leg standing positions; the cartilage width; and the length of the osteophyte in each of the four sites were recorded. For each examiner, the average value was also calculated.

Intra-observer reliability for evaluating the structures was assessed using data from both sessions for each examiner, and the mean measurements from the two sonographers were compared to calculate inter-observer reliability.

### 2.4. Statistical Analysis

Statistical analysis was conducted using Microsoft Excel and SPSS version 26. Inter-observer reliability was evaluated using ICCs based on a two-way random-effects model and absolute agreement ICC (2,1). ICC values were interpreted as poor (0.50), moderate (0.50–0.70), good (0.75–0.90) or excellent (>0.90). Measurement precision was assessed by calculating the standard error of measurement (SEM) SEM = SD × $\sqrt{1-ICC}$ (SD—Standard deviation of baseline score) and the minimum detectable change at the 95% confidence level (MDC95) MDC95 = 1.96 × √2 × SEM. Differences in meniscal extrusion between the supine and single-leg-stand weight-bearing positions were compared with the MDC95 to determine whether the observed changes exceeded measurement errors and represented true load-related variation. Agreement between examiners was further evaluated using Bland–Altman analysis, including calculation of the mean difference (bias) and 95% limits of agreement (LoA), defined as the mean difference ± 1.96 × standard deviation of the differences. Statistical significance was set at *p* < 0.05. Agreement for ordinal osteophyte grading was assessed using Cohen’s kappa statistics and repeated assessments. Kappa values were interpreted according to Landis and Koch’s criteria, and 95% CI intervals were calculated using asymptotic standard error.

Intra-rater reliability was evaluated separately for each examiner using ICCs from a two-way mixed-effects model with absolute agreement (ICC (3,1)). The two repeated measurements made by each examiner were included in the analysis. Measurement error was quantified using the SEM, and the minimal detectable change at the MCD95 was calculated accordingly. Differences in examination time between repeated measurements and between examiners were analyzed using paired-samples tests. Statistical significance was set at *p* < 0.05.

## 3. Results

### 3.1. Demographic Characteristics of the Analyzed Group of Patients

The study group included 25 patients diagnosed with KOA (ACR criteria), aged 40–74 years [28]. A total of 50 knee joints were assessed according to the protocol established before the study began. The population characteristics, including age, BMI, time since diagnosis, pain level on the NRS and K-L stage are presented in Table 1 and Table 2.

The quality of the US image achieved was quantified by each examiner on a scale of 1 (good), 2 (medium) or 3 (low), as described in Table 3.

Most US examinations were classified as good or acceptable quality, while poor-quality images were uncommon.

### 3.2. Ultrasound Assessment of Medial and Lateral Meniscal Extrusion

The number of patients with medial meniscal protrusion > 3 mm increased with loading. For the first evaluator, this was identified in 30 of 50 knees in the supine position, 45 in the standing position and 49 in the single-leg standing position. Similar results were observed for the second evaluator (27, 44 and 48 of 50 knees, respectively).

For both evaluators, medial meniscal extrusion increased with increasing loading. Mean values for Examiner 1 were 3.220 mm in the supine position, 3.746 mm during bipedal standing and 4.219 mm during single-leg standing, whereas Examiner 2 obtained corresponding measurements of 3.194 mm, 3.682 mm and 4.266 mm.

The mean lateral meniscal extrusion values progressively increased from the supine to bipedal standing and single-leg standing positions for both evaluators. For Examiner 1, the mean values were 3.53 mm in supine position, 4.046 mm in bipedal standing and 4.602 mm in single-leg standing, while Examiner 2 recorded corresponding values of 3.418 mm, 3.904 mm and 4.402 mm, respectively.

#### 3.2.1. Intra-Rater Reliability of Ultrasound Assessment of Medial and Lateral Meniscal Extrusion

Intra-rater reliability was evaluated separately for each examiner using the ICC derived from a two-way mixed-effects model with absolute agreement (ICC (3,1)).

The first evaluator ranged from 0.77 to 0.88. For the medial meniscus, ICC values range from 0.77 to 0.87, indicating good repeatability. For the lateral meniscus, ICC values were slightly higher, from 0.81 to 0.88, reflecting good reliability as well, as seen in Table 4.

SEM values ranged from 0.25 mm to 0.43 mm, and MDC95 values ranged from 0.70 mm to 1.18 mm. The largest measurement error was observed in the medial meniscus during unipedal standing, and the lowest measurement was in the lateral meniscus in the bipedal position.

Intra-rater reliability for Evaluator 2 ranged from 0.63 to 0.92. For the lateral meniscus, ICC values ranged from moderate (0.63 in unipedal standing) to excellent (0.92 in bipedal), indicating a slight heterogeneity across positions, while the repeatability was good in most positions, as Table 5 describes.

SEM values ranged from 0.21 mm to 0.59 mm, while MDC95 values ranged from 0.59 mm to 1.63 mm.

#### 3.2.2. Inter-Rater Reliability of Ultrasound Assessment of Medial and Lateral Meniscal Extrusion

Inter-rater reliability was assessed using a two-way random-effects model (ICC (2,1), absolute agreement). For the medial meniscus, ICC values ranged from 0.79 to 0.88 across positions, indicating good agreement. For the lateral meniscus, ICC values ranged from 0.72 to 0.87, likewise demonstrating moderate to good reliability.

SEM values ranged from 0.26 mm to 0.47 mm, and MDC95 ranged from 0.72 mm to 1.32 mm. The lowest measurement error was observed for medial meniscal extrusion in the supine position (SEM = 0.26 mm; MDC95 = 0.72 mm), whereas the highest was found for lateral meniscal extrusion in the unipedal position (SEM = 0.47 mm; MDC95 = 1.32 mm). All this data can be found in Table 6.

The Bland–Altman plot shows minimal systematic bias between raters between repeated measurements. For the medial meniscus, mean differences ranged from −0.01 to 0.02, with 95% limits of agreement (LoA) ranging from −0.92 to 0.92 mm across the three examination positions. Lateral meniscus measurements showed a bias of 0.12 to 0.2 mm, with a wider 95% LoA from −1.08 to 1.48 mm. Overall, the agreement is acceptable, with no evident proportional bias. Consistent with these findings, inter-rater reliability analysis demonstrated good agreement across all measurement conditions, with ICCs ranging from 0.72 to 0.88. Measurement error was low, as reflected by SEM values between 0.26 and 0.47, yielding an MDC 95 threshold ranging from 0.76 to 1.48 mm that can be interpreted as a meaningful assessment of meniscal protrusion. Detailed reliability values are presented in the Supplementary Materials.

### 3.3. Reliability of Ultrasound Assessment of Femoral Cartilage

#### 3.3.1. Intra-Rater Reliability of Ultrasound Assessment of Femoral Cartilage

Intra-rater reliability was good for both examiners across all cartilage regions. For Examiner 1, ICC values were equal to 0.76 for all of the three compartments, with SEM values between 0.20 and 0.30 mm and MDC95 values ranging from 0.55 to 0.82 mm. Examiner 2 showed comparable reliability; however, the values were lower than 0.75, showing rather moderate reliability. Measurement error remained low, with SEM values ranging from 0.24 to 0.34 mm and MDC95 values between 0.67 and 0.94 mm. The inter-rater results are widely shown in Table 7.

#### 3.3.2. Inter-Rater Reliability of Ultrasound Assessment of Femoral Cartilage

Inter-rater reliability analysis demonstrated good agreement for lateral cartilage thickness measurements, moderate agreement for medial cartilage and low agreement for the trochlear site. ICC values ranged from 0.48 to 0.82. The highest reliability was observed for lateral cartilage (ICC = 0.82; 95% CI: 0.70–0.89), followed by medial cartilage (ICC = 0.70; 95% CI: 0.53–0.82), while trochlear cartilage showed lower reliability (ICC = 0.43; 95% CI: 0.24–0.66). Measurement error analysis revealed SEM values between 0.20 and 0.41 mm, corresponding to MDC95 values ranging from 0.54 to 1.14 mm.

Bland–Altman analysis demonstrated minimal systematic bias between examiners, with mean differences close to zero (−0.124 to −0.10 mm). The 95% limits of agreement were relatively narrow for medial and lateral cartilage measurements, whereas wider limits were observed for trochlear cartilage, indicating increased variability in this region. Detailed agreement data are presented in Table 8, and Bland–Altman plots are shown in Supplementary Material Figure S1.

### 3.4. Reliability of Ultrasound Assessment of the Osteophytes

#### 3.4.1. Intra-Rater Reliability of Ultrasound Assessment of the Osteophytes

Intra-rater reliability analyses demonstrated excellent agreement for osteophyte measurements across all evaluated compartments for both examiners. ICC values ranged from 0.95 to 0.99, indicating excellent repeatability of measurements.

For Examiner 1, ICC values ranged between 0.95 and 0.98. The highest reliability was observed for tibial lateral osteophytes (ICC = 0.98; 95% CI: 0.96–0.99), and both tibial and femoral medial osteophytes (ICC = 0.97; 95% CI: 0.95–0.98). Furthermore, SEM ranges from 0.17 to 0.28, as shown in Table 9.

For Examiner 2, ICC values ranged from 0.91 to 0.99, confirming comparable repeatability. Measurement error remained low, with SEM values ranging from 0.18 to 0.53 mm and corresponding MCD95 values between 0.50 and 1.47 mm.

Overall, minimal measurement variability was observed between repeated assessments, supporting excellent intra-observer consistency. Detailed results are presented in Table 10.

#### 3.4.2. Inter-Rater Reliability of Ultrasound Assessment of the Osteophytes

Intra-rater agreement for osteophyte grading showed substantial agreement across all evaluated compartments. Cohen’s kappa values ranged from 0.719 to 0.868. High agreement was observed for femoral medial (k = 0.85; 95% CI: 0.72–0.98), femoral lateral (k = 0.83; 85% CI: 0.69–0.97) and tibial lateral osteophytes (k = 0.72; 95% CI: 0.73–1.00). Substantial agreement was found for tibial medial osteophytes (k = 0.72; 95% CI: 0.56–0.88). Detailed results are shown in Table 11.

### 3.5. Time Needed for Evaluation

The time taken by each of two evaluators to perform the examination in the three different patients’ positions was recorded. The time required for each assessment by each examiner is shown in Table 12.

The mean examination time ranged from 5.76 to 7.40 min. No significant differences were observed between examiners during the first (*p* = 0.474) or second (*p* = 0.511) assessments. Within-examiner comparison showed no significant difference between repeated measurements for Examiner 1 (*p* = 0.097). However, Examiner 2 showed a significantly shorter examination time during the second assessment compared with the first evaluation (mean difference 0.46 min, *p* = 0.002). The data are described in Table 13.

## 4. Discussion

### 4.1. Main Findings of the Study

Measurement error analysis demonstrated relatively low SEM and MDC95 values across the evaluated structures, supporting the precision of the US measurements obtained using the standardized protocol.

Regarding meniscal extrusion in particular, the low SEM values observed suggest limited variability between repeated measurements and support US’s ability to detect small positional changes. Reliability ranged from moderate to good, with higher ICC observed in the supine position and slightly lower values in weight-bearing positions. Compared with previous studies reporting similarly moderate to excellent reliability of meniscal displacement assessment, our findings align with the reported values [29,30]. Overall inter-rater reliability was slightly lower in weight-bearing positions, which may reflect greater reliability in posture, joint loading and probe positioning. In contrast, the unipedal position showed greater reliability in medial meniscus extrusion measurements. These findings may be explained by the increased load on the medial compartment during single-leg stance, which stabilizes the medial meniscus and clarifies its borders identified on MSUS. Additionally, the medial meniscus’s lower mobility may reduce positional variability under these conditions, resulting in more consistent measurements [31]. Previous studies have also shown that meniscal extrusion is influenced by loading conditions and changes in meniscal position, with dynamic ultrasound showing that meniscal extrusion varies, especially in pathological knees [13,32]. Our findings are consistent with previous studies demonstrating that ultrasound is a reliable method for assessing medial meniscal extrusion, with good agreement with Magnetic Resonance Imaging (MRI) when standardized measurement techniques are applied. A recent systematic review reported a strong correlation between US and MRI, demonstrating high specificity and sensitivity for ultrasound and high diagnostic accuracy [33]. Furthermore, quantitative assessment showed substantial agreement between the two modalities (ICC: 0.70–0.73), and moderate to excellent intra- and inter-reliability was found for meniscal displacement on ultrasound evaluation (0.904 and 0.942, respectively) [33,34].

Intra-rater reliability for the two examiners was good across all positions, with most of the ICC values exceeding 0.80, and showed high reproducibility for meniscal displacement assessment, being consistent with previous studies [35].

Currently available studies use heterogeneous US techniques for assessing meniscal extrusion, with variations in patient positioning, anatomical landmarks and acquisition methods. This methodological variability may contribute to differences in reported extrusion values and to reproducibility across studies. Farivar et al. recently proposed a standardized US technique for assessing meniscal extrusion in patients with posterior medial root meniscal tears, further highlighting the importance of consistent acquisition methods [15]. Compared with this protocol, which evaluated extrusion with the knee flexed at 30 degrees in a standing position, the present study assessed two loading positions to provide additional functional information on meniscal behavior under weight-bearing conditions.

Cartilage assessment demonstrated generally good reproducibility, aligning with previous studies reporting likewise moderate to good reliability; only one value fell within the range indicative of low repeatability. Our findings were broadly comparable, with slightly higher values in the medial and lateral compartments [29]. Agreement analysis showed narrower limits of agreement for the medial and lateral condyles. Wider limits of agreement were observed for the trochlear cartilage, in line with previous reports suggesting that ultrasound assessment of femoral cartilage may show variability across compartments, with the trochlear level being related to the more complex anatomy [36,37]. Our findings are consistent with previous studies, which show good agreement in cartilage assessment using a semi-quantitative (1–3) grading system [38]. The slightly higher reliability observed in our study may reflect the use of continuous measurements, which reduces observer dependency.

For the osteophytes, the ICC values observed in this study for all tibio-femoral osteophyte sites are consistent with the higher end of previous reports, indicating very high measurement consistency. This may be partly explained by the use of a standardized protocol, including a semi-quantitative grading approach, which may have reduced variability between repeated measurements [16,36]. Furthermore, osteophytes are bony structures with well-defined borders which facilitate their identification and increase inter-rater reproducibility.

### 4.2. Reliability and Agreement Considerations

The improved reproducibility observed in our cohort may be explained by the use of a standard internal protocol within the same institution, with the same imaging US machine and settings, including consistent acquisition techniques and similar experience among the two evaluators. These factors are known to reduce variability. The selection of patients with moderate KOA (K-L grades 2–3) may have influenced measurement consistency, as this stage may offer a balance between detectable structural changes and preserved anatomical landmarks [36,39]. However, this level of standardization may also limit the generalizability of the findings to other settings.

The predominance of good and acceptable image quality may have contributed to the high reproducibility observed in this study. Minor differences in image ratings among evaluators likely reflect the operator-dependent nature of MSUS, as in current EULAR recommendations.

Although ultrasound image quality was systematically recorded and most examinations were rated as good or medium, only a limited number of low-quality images were obtained. This reflects the implementation of a standardized protocol and the experience of the operators. However, image quality remains a potential source of variability, particularly for the assessment of trochlear cartilage. Similarly, other patient-related factors such as body mass index, gender and radiographic severity (K-L grade) may influence measurement reproducibility. The study population had a mean BMI of 26.0 ± 4.06 kg/m^2^ with relatively few obese individuals and consisted predominantly of female patients (72%). Moreover, only patients with K-L grades 2–3 were included by design, ensuring measurable structural changes while avoiding end-stage disease. Nevertheless, these characteristics should be considered when interpreting the reproducibility results and generalizing the findings to broader populations with higher BMI or different disease stages.

### 4.3. Clinical Implications

Different thresholds for pathological meniscal extrusion have been proposed in the literature, particularly in US-based studies. Although the 3 mm threshold was initially derived from MRI studies, it has been widely adopted in US research and remains a commonly accepted criterion for pathological medial meniscal extrusion. The magnitude of meniscus displacement is clinically relevant, particularly when it exceeds this limit. On MRI assessment, this reflects significant pathology and is essential for establishing an accurate diagnosis and an appropriate therapeutic approach [12]. Some studies have demonstrated that meniscal extrusion increases under weight-bearing conditions, indicating that extrusion may become more evident in functional positions, consistent with the present study’s findings [39,40].

A higher number of medial extrusions exceeding the commonly used 3 mm threshold was observed under weight-bearing conditions than in the supine position. However, although a threshold of >3 mm is used in both ultrasound and MRI studies to define medial meniscal extrusion, its clinical interpretation remains debated, and the relationship between load-dependent extrusion and disease severity or symptoms has not yet been fully established. In contrast, no universally accepted pathological threshold currently exists for lateral meniscal extrusion [12].

These findings highlight the potential value of dynamic ultrasound assessment, as structural changes that become apparent only under loading conditions may not be detected during conventional non-weight-bearing examinations. Although this functional assessment adds complementary information to the proposed ultrasound protocol, the clinical significance of load-dependent meniscal extrusions requires further investigation.

### 4.4. Study Limitations, Strengths and Future Research Projects

This study has several limitations. First, the relatively small sample size and single-center design may limit the generalizability of the results. Second, the use of a standardized protocol and evaluators with similar experience may contribute to the high level of reproducibility observed but may also limit the applicability of the findings to other clinical settings. The use of a semi-quantitative scale, which improves reproducibility by reducing the impact of minor variations between measurements, may limit sensitivity to subtle differences in osteophyte size. Only a small number of examinations (one for the first evaluator and two for the second) were rated as poor quality, limiting the assessment of reproducibility under suboptimal conditions. Furthermore, selecting patients with moderate osteoarthritis (K-L grades 2–3) limits the generalizability of the results to other disease stages. Ultrasound remains an operator-dependent method, and variability may increase under different examination conditions. In addition, the lack of a systematic assessment of synovitis and synovial hypertrophy, and the absence of a direct comparison with MRI constitute limitations of this study.

The absence of a comparison with MRI, the reference imaging modality for evaluating cartilage and meniscal abnormalities, is another limitation [34]. Although in vivo dynamic MRI studies have demonstrated load-dependent changes in meniscal position, their use remains largely limited to research settings and is not routinely available in clinical practice [31]. Nevertheless, the objective of this study was to establish the diagnostic validity of MSUS but also to assess the reproducibility of a standardized US protocol under different loading conditions.

Another aspect to consider when interpreting the findings is that both knees of the same participant were included in the analysis. As this study aimed to evaluate the reproducibility of ultrasound measurements at the knee level, each knee was analyzed as an individual anatomic unit. Nevertheless, the inclusion of bilateral knees may represent a potential source of within-subject correlation, which should be considered when interpreting the results. Future studies including larger cohorts may address this aspect using statistical approaches designed for clustered data.

The present study should also be interpreted in the context of its pilot design. The relatively small sample size and single-center setting may limit the generalizability of the findings. Future multicentric studies involving larger and heterogeneous populations are needed to further validate the reproducibility and clinical applicability of the proposed ultrasound protocol.

Additionally, although image quality was systematically recorded, its direct influence on reproducibility metrics, as well as the potential moderating effects of BMI, gender and radiographic severity (K-L grade), were not subjected to subgroup analysis in this pilot study. Future larger-scale investigations should address these interactions to further refine the applicability of the proposed protocol across diverse patient populations.

This study has several strengths, including the use of the same equipment, settings and evaluators with comparable experience, which helped reduce variability and achieve high reproducibility. Moreover, the ultrasound evaluation was performed in different positions, including weight-bearing positions, which have been understudied in prior research.

Regarding the results of the present study, future research could build on this observation by analyzing patients across all stages of KOA. Moreover, the relationships between clinical, functional and ultrasound data need clarification, and longitudinal studies following patients undergoing different treatments over time would be valuable. In addition, comparisons between US results and MRI, the gold-standard method, could help develop standardized protocols for US assessment of the knee under loading conditions and lead to the method’s validation and clinical application.

## 5. Conclusions

The proposed ultrasound protocol demonstrated good reproducibility in assessing cartilage thickness, osteophytes and meniscal extrusion across different loading conditions, supporting its use as a standardized assessment method. However, the clinical significance of load-dependent meniscal extrusion remains to be established. Further prospective studies are needed to determine its relationship with structural disease severity, clinical symptoms and functional outcomes in patients with knee osteoarthritis.

## Figures and Tables

**Table 1:** Descriptive statistics 1.

	Mean	S.D.
AGE	57.56	9.75
BMI	26.00	4.06
NRS—Right knee	4.24	2.58
NRS—Left knee	4.08	2.85
Time since diagnosis (years)	5.08	3.01

S.D. (Standard deviation), BMI (Body mass index), NRS (Numeric Rating Scale), K-L (Kellgren–Lawrence).

**Table 2:** Descriptive statistics 2.

Variable	Category	n	%
Gender	Female	18	72
	Male	7	28
K-L—Right knee	2	15	60
	3	10	40
K-L—Left knee	2	17	68
	3	8	32

K-L (Kellgren–Lawrence), n (number).

**Table 3:** Descriptive statistics of image quality.

Variable	Category	n	%
Image quality E1	1	12	48
	2	12	48
	3	1	4
Image quality E2	1	9	36
	2	14	56
	3	2	8

E1 (Examiner 1), E2 (Examiner 2), n (number).

**Table 4:** Intra-rater reliability of meniscus protrusion for Examiner 1.

Meniscus	Position	N (Knees)	ICC (3,1)	95% CI (Lower–Upper)	SD (mm)	SEM (mm)	MDC95 (mm)
Medial	Supine	50	0.87	0.78–0.92	0.84	0.30	0.84
	Bipedal	50	0.86	0.77–0.92	0.73	0.27	0.75
	Unipedal	50	0.77	0.62–0.86	0.89	0.43	1.18
Lateral	Supine	50	0.81	0.68–0.89	0.85	0.32	0.91
	Bipedal	50	0.88	0.80–0.93	0.85	0.25	0.70
	Unipedal	50	0.84	0.73–0.90	0.95	0.35	0.98

N (number), ICC (intraclass correlation coefficient), 95% CI (95% confidence interval), SD (standard deviation), SEM (standard error of measurement), MCD95 (95% confidence level).

**Table 5:** Intra-rater reliability of meniscus protrusion for Examiner 2.

Meniscus	Position	N (Knees)	ICC (3,1)	95% CI (Lower–Upper)	SD (mm)	SEM (mm)	MDC95 (mm)
Medial	Supine	50	0.71	0.52–0.82	0.74	0.40	1.11
	Bipedal	50	0.85	0.76–0.91	0.73	0.28	0.77
	Unipedal	50	0.88	0.79–0.93	0.87	0.30	0.84
Lateral	Supine	50	0.82	0.71–0.90	0.81	0.34	0.94
	Bipedal	50	0.92	0.87–0.96	0.77	0.21	0.59
	Unipedal	50	0.63	0.43–0.77	0.97	0.59	1.63

N (number), ICC (intraclass correlation coefficient), 95% CI (95% confidence interval), SD (standard deviation), SEM (standard error of measurement), MCD95 (95% confidence level).

**Table 6:** Inter-rater reliability of meniscus protrusion between examiners.

Meniscus	Position	N	ICC (2,1)	95% CI	SD (mm)	SEM (mm)	MDC95 (mm)	Bias (mm)	LLoA	ULoA
Medial	Supine	50	0.88	0.79–0.93	0.75	0.26	0.72	0.02	−0.71	0.76
	Bipedal	50	0.79	0.66–0.87	0.70	0.32	0.89	−0.01	−0.92	0.89
	Unipedal	50	0.86	0.76–0.92	0.85	0.32	0.89	0.01	−0.89	0.92
Lateral	Supine	50	0.87	0.80–0.93	0.79	0.27	0.74	0.12	−0.60	0.84
	Bipedal	50	0.75	0.60–0.85	0.79	0.39	1.06	0.13	−0.94	1.20
	Unipedal	50	0.72	0.55–0.83	0.90	0.47	1.32	0.20	−1.08	1.48

N (number), ICC (intraclass correlation coefficient), 95% CI (95% confidence interval), SD (standard deviation), SEM (standard error of measurement), MCD95 (95% confidence level), LLoA (lower limit of agreement), ULoA (upper limit of agreement).

**Table 7:** Intra-rater reliability of cartilage width for the two examiners.

Cartilage	N (Knees)	ICC (3,1)	95% CI (Lower–Upper)	SD (mm)	SEM (mm)	MDC95 (mm)
Medial E1	50	0.76	0.60–0.85	0.41	0.20	0.56
Lateral E1	50	0.76	0.60–0.85	0.48	0.24	0.66
Trochlear E1	50	0.76	0.62–0.86	0.61	0.30	0.82
Medial E2	50	0.71	0.54–0.82	0.45	0.24	0.67
Lateral E2	50	0.62	0.41–0.76	0.55	0.34	0.94
Trochlear E2	50	0.73	0.57–0.84	0.56	0.29	0.81

N (number), ICC (intraclass correlation coefficient), 95% CI (95% confidence interval), SD (standard deviation), SEM (standard error of measurement), MCD95 (95% confidence level), E1 (examiner 1), E2 (examiner 2).

**Table 8:** Inter-rater reliability of cartilage width between examiners.

Cartilage	N	ICC (2,1)	95% CI (Lower–Upper)	SD (mm)	SEM (mm)	MDC95 (mm)	Bias (mm)	LLoA	ULoA
Medial	50	0.70	0.53–0.82	0.40	0.22	0.6	−0.01	−0.62	0.59
Lateral	50	0.82	0.70–0.89	0.47	0.20	0.55	−0.04	−0.60	0.51
Trochlear	50	0.48	0.24–0.66	0.57	0.41	1.14	−0.12	−1.25	1.01

N (number), ICC (intraclass correlation coefficient), 95% CI (95% confidence interval), SD (standard deviation), SEM (standard error of measurement), MCD95 (95% confidence level), LLoA (lower limit of agreement), ULoA (upper limit of agreement).

**Table 9:** Intra-rater variability in osteophyte dimensions for Examiner 1.

Bone	Osteophyte	N (Knees)	ICC (3,1)	95% CI (Lower–Upper)	SD (mm)	SEM (mm)	MDC95 (mm)
Femoral	Medial	50	0.97	0.95–0.98	1.73	0.3	0.83
Tibial		50	0.97	0.95–0.98	1.59	0.28	0.78
Femoral	Lateral	50	0.95	0.97–0.99	1.92	0.23	0.65
Tibial		50	0.98	0.96–0.99	1.18	0.17	0.49

N (number), ICC (intraclass correlation coefficient), 95% CI (95% confidence interval), SD (standard deviation), SEM (standard error of measurement), MCD95 (95% confidence level).

**Table 10:** Intra-rater variability in osteophyte dimensions for Examiner 2.

Bone	Osteophyte	N (Knees)	ICC (3,1)	95% CI (Lower–Upper)	SD (mm)	SEM (mm)	MDC95 (mm)
Femoral	Medial	50	0.91	0.84–0.95	1.74	0.53	1.47
Tibial		50	0.98	0.97–0.99	1.61	0.20	0.55
Femoral	Lateral	50	0.99	0.98–0.99	1.89	0.21	0.57
Tibial		50	0.98	0.96–0.99	1.25	0.18	0.50

N (number), ICC (intraclass correlation coefficient), 95% CI (95% confidence interval), SD (standard deviation), SEM (standard error of measurement), MCD95 (95% confidence level).

**Table 11:** Cohen’s Kappa for inter-rater variability in osteophyte dimensions.

Bone	Osteophyte	N (Knees)	κ	SE	95% CI Low	95% CI Up	Interpretation
Femoral	Medial	50	0.85	0.06	0.72	0.97	Almost perfect
Tibial		50	0.71	0.08	0.56	0.87	Substantial agreement
Femoral	Lateral	50	0.83	0.06	0.69	0.96	Almost perfect
Tibial		50	0.86	0.07	0.73	1.00	Almost perfect

N (number), k (Cohen’s kappa statistic coefficient), SE (Standard error), 95% CI (95% confidence interval).

**Table 12:** Descriptive statistics of evaluation time.

	Mean	S.D.
T E1 a (minutes)	6.21	1.08
T E1 b (minutes)	5.90	0.87
T E2 a (minutes)	6.40	1.43
T E2 b (minutes)	5.76	1.15

S.D. (Standard deviation), T (evaluation time), E1 (Examiner 1), E2 (Examiner 2), a (1st evaluation), b (2nd evaluation).

**Table 13:** Examination time for the two examiners.

Paired Samples Test		Mean	Lower 95% CI	Upper 95% CI	t	*p*
Pair 1	T E1 a–T E1 b	0.31	−0.06	0.68	1.727	0.097
Pair 2	T E2 a–T E2 b	0.64	0.26	1.01	3.562	0.002
Pair 3	T E1 a–T E2 a	−0.18	−0.72	0.34	−0.728	0.474
Pair 4	T E1 b–T E2 b	0.13	−0.29	0.56	0.667	0.511

CI (95% confidence interval), t (t-value), *p* (*p*-value), a- first evaluation, b- second evaluation.

## Data Availability

The original contributions presented in this study are included in the article/its Supplementary Materials. Further inquiries can be directed to the corresponding authors.

## Supplementary Materials

The following supporting information can be downloaded at https://www.mdpi.com/article/10.3390/medicina62071292/s1, Figure S1: Bland–Altman graphs. (A–F) Band-Altmann plots—inter-rater agreement for the medial and lateral meniscal protrusion across three conditions. (G–I) Band-Altmann plots—inter-rater cartilage width measurements. Red line—mean bias; blue and yellow lines—upper and lower limits of agreement.; Figure S2: Informed consent form; Figure S3: Schematic illustration of standardized musculoskeletal examining protocol.

## Abbreviations

The following abbreviations are used in this manuscript:

95%CI	95% confidence interval
a	1st evaluation
ACR	American College of Rheumatology
b	2nd evaluation
BRST	Balneal and Rehabilitation Sanatorium of Techirghiol
BMI	Body mass index
E1	Examiner 1
E2	Examiner 2
EULAR	European League Against the Rheumatism
ICC	Intraclass correlation coefficient
ICCs	Intraclass correlation coefficients
K-L	Kellgren–Lawrence
KOA	Knee osteoarthritis
LLoA	Lower limit of agreement
LoA	Limits of agreement
MDC95	Minimal detectable chance 95% confidence level
MSUS	Musculoskeletal ultrasound
MRI	Magnetic Resonance Imaging
N	Number
NRS	Numeric Rating Scale
OA	Osteoarthritis
OMERACT	Outcome Measures in Rheumatology
*p*	*p*-value
SD	Standard deviation
SDx	Standard deviation of baseline score
SE	Standard error
SEM	Standard error of measurement
t	t-value
T	Evaluation time
ULoA	Upper limit of agreement
US	Ultrasound
VAG	Vibroarthrography
X-ray	Radiography
