# Supplementary material for: Musculoskeletal Ultrasound Reliability in Knee Osteoarthritis: A Pilot Study of Cartilage Thickness, Osteophytes and Weight-Bearing Meniscal Extrusion
Source: Medicina (Kaunas). 2026 Jul 4;62(7):1292. doi: 10.3390/medicina62071292 (PMC13413949; doi:10.3390/medicina62071292)
Supplement: Supplementary file 1 [file medicina-62-01292-s001.zip › Figure S1. Band-Altman graphs.pdf]

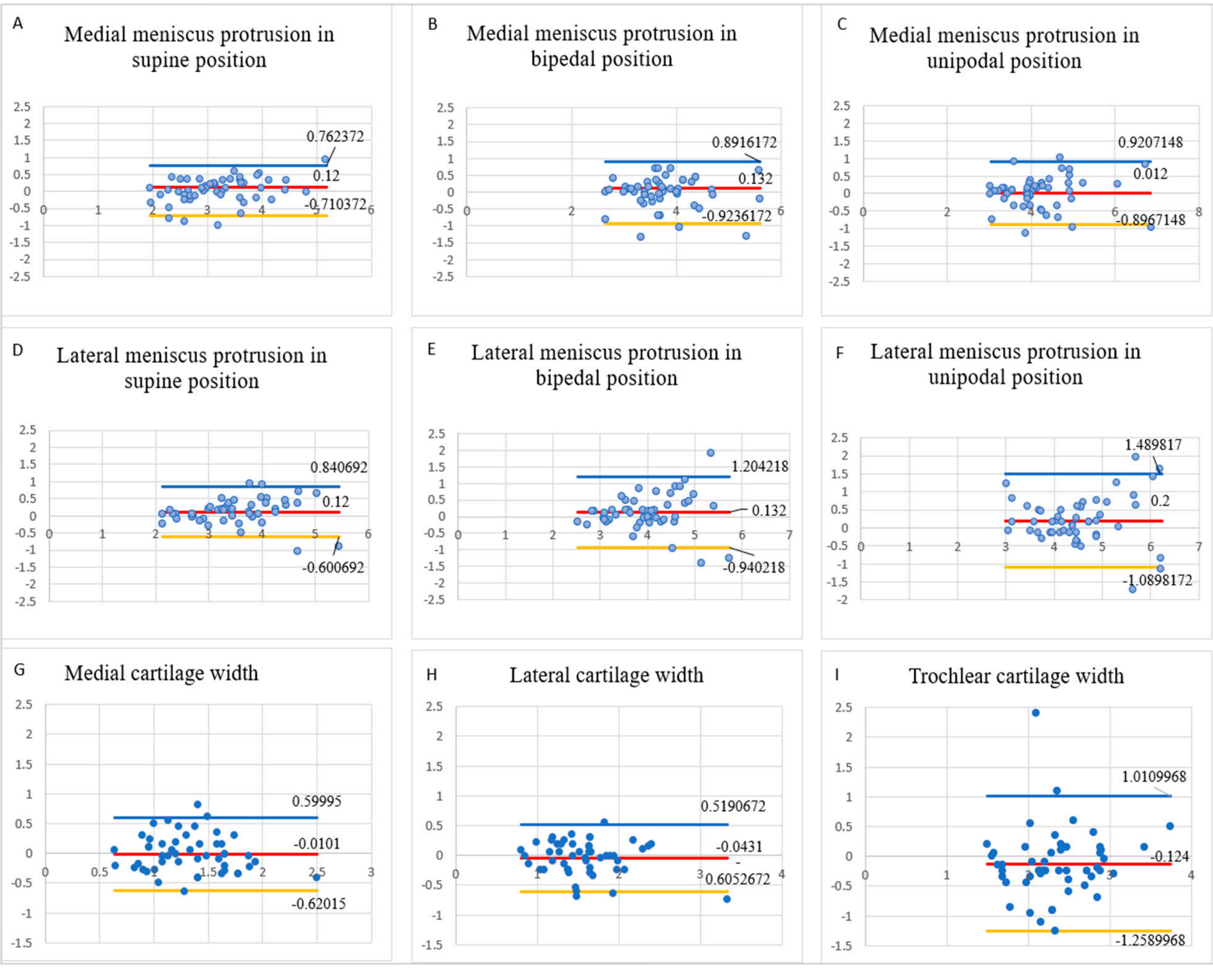


**Figure S1. Band-Altman graphs**

(A-F) Band-Altmann plots- inter-rater agreement for the medial and lateral meniscal protrusion across three conditions. (G-I) Band-Altmann plots – inter-rater cartilage width measurements. Red line - mean bias; blue and yellow lines - upper and lower limits of agreement.
