# Supplementary material for: Musculoskeletal Ultrasound Reliability in Knee Osteoarthritis: A Pilot Study of Cartilage Thickness, Osteophytes and Weight-Bearing Meniscal Extrusion
Source: Medicina (Kaunas). 2026 Jul 4;62(7):1292. doi: 10.3390/medicina62071292 (PMC13413949; doi:10.3390/medicina62071292)
Supplement: Supplementary file 1 [file medicina-62-01292-s001.zip › Figure S2 - Informed consent form.pdf]

SANATORIUL BALNEAR ŞI DE RECUPERARE TECHIRGHIOL

MINISTRY OF HEALTH
BALNEAL SANATORIUM OF TECHIRGHIOL

Bd. Dr. Victor Climescu, nr 34-40; Tel: 0241 481 711; Fax: 0241 735 705; Web.www.sbtghiol.ro; e-mail: sbtghiol@sbtghiol.ro

CONSIMȚĂMÂNTUL INFORMAT AL PACIENTULUI

Subsemnatul .... , CNP .... Domiciliat în localitatea ...., Județul ...., Sectorul ...., Strada..., Număr ...., Bloc ...., Scara ...., Etaj ...., Apartament .... Telefon ...., Având Diagnosticul ....

- Sunt de acord cu participarea la studiul “ **FIABILITATEA ECOGRAFIEI MUSCULOSCHELETALE ÎN OSTEOARTRITA GENUNCHIULUI: STUDIU PILOT PRIVIND GROSIMEA CARTILAJULUI, OSTEOFITELE ȘI EXTRUZIA MENISCALĂ ÎN CONDIȚII DE SPRIJIN PE MEMBRUL INFERIOR**”, cu efectuarea tuturor examenelor clinice și de laborator necesare și efectuarea tratamentului recomandat de medical curant;
- Menționez că am citit și am înțeles formularul de informare al acestui studiu;
- Menționez că particip voluntar la acest studiu, am posibilitatea de a mă retrage oricând doresc fără a explica motivele și fără ca retragerea din studiu să îmi afecteze îngrijirea terapeutică ulterioară;
- Permit accesul la datele mele medicale, fără încălcarea obligațiilor de confidențialitate, celor care efectuează studiul, comitetelor de etică medicală și autorităților de reglementare din domeniu;
- Sunt de acord să urmez instrucțiunile medicului curant, să raspund la întrebări și să semnalez în timp util orice manifestare clinică survenită pe parcursul efectuării tratamentului;
- Sunt de acord ca datele obținute să fie prelucrate matematic și interpretate statistic în vederea publicării unor articole în baze de date naționale și internaționale, cu păstrarea confidențialității.

Semnătură pacient:
Semnătură medic curant:
Semnătură martor:

Unitatea Sanitară unde se desfășoară tratamentul: Sanatoriul Balnear și de Recuperare Techirghiol

MINISTRY OF HEALTH
BALNEAL SANATORIUM OF TECHIRGHIOL

Bd. Dr. Victor Climescu, nr 34-40; Tel: 0241 481 711; Fax: 0241 735 705; Web.www.sbtghiol.ro; e-mail: sbtghiol@sbtghiol.ro

PATIENT'S INFORMED CONSENT

The undersigned.. ...., CNP .... Domiciled in the locality ...., County.. ...., Sector..., The street.., Number................................... , Block ...., Stairs ...., Floor ...., Apartment .... Telephone ...., Having the Diagnosis ....

I agree to participate in the study **“MUSCULOSKELETAL ULTRASOUND RELIABILITY IN KNEE OSTEOARTHRITIS: A PILOT STUDY OF CARTILAGE THICKNESS, OSTEOPHYTES AND WEIGHT-BEARING MENISCAL EXTRUSION”,** to perform all the necessary clinical and laboratory examinations and perform the treatment recommended by the attending physician;

- I mention that I have read and understood the information form of this study;
- I mention that I am voluntarily participating in this study, I can withdraw at any time without explaining the reasons and without the withdrawal from the study affecting my subsequent therapeutic care;
- I allow access to my medical data, without breach of confidentiality obligations, to those conducting the study, medical ethics committees and regulatory authorities in the field;
- I agree to follow the instructions of the attending physician, answer questions and to report promptly any clinical manifestation occurring during treatment;
- I agree that the data obtained will be mathematically processed and statistically interpreted to publish articles in national and international databases, preserving confidentiality.

Patient signature:
Attending physician signature:
Witness signature:

The Health Unit where the treatment is carried out:
Techirghiol Balneal and Rehabilitation Sanatorium
