# Supplementary material for: Musculoskeletal Ultrasound Reliability in Knee Osteoarthritis: A Pilot Study of Cartilage Thickness, Osteophytes and Weight-Bearing Meniscal Extrusion
Source: Medicina (Kaunas). 2026 Jul 4;62(7):1292. doi: 10.3390/medicina62071292 (PMC13413949; doi:10.3390/medicina62071292)
Supplement: Supplementary file 1 [file medicina-62-01292-s001.zip › Figure S3 - Schematic illustration of standardized musculoskeletal examining protocol (1).pdf]

Figure 3. Ultrasound positioning and measurement technique

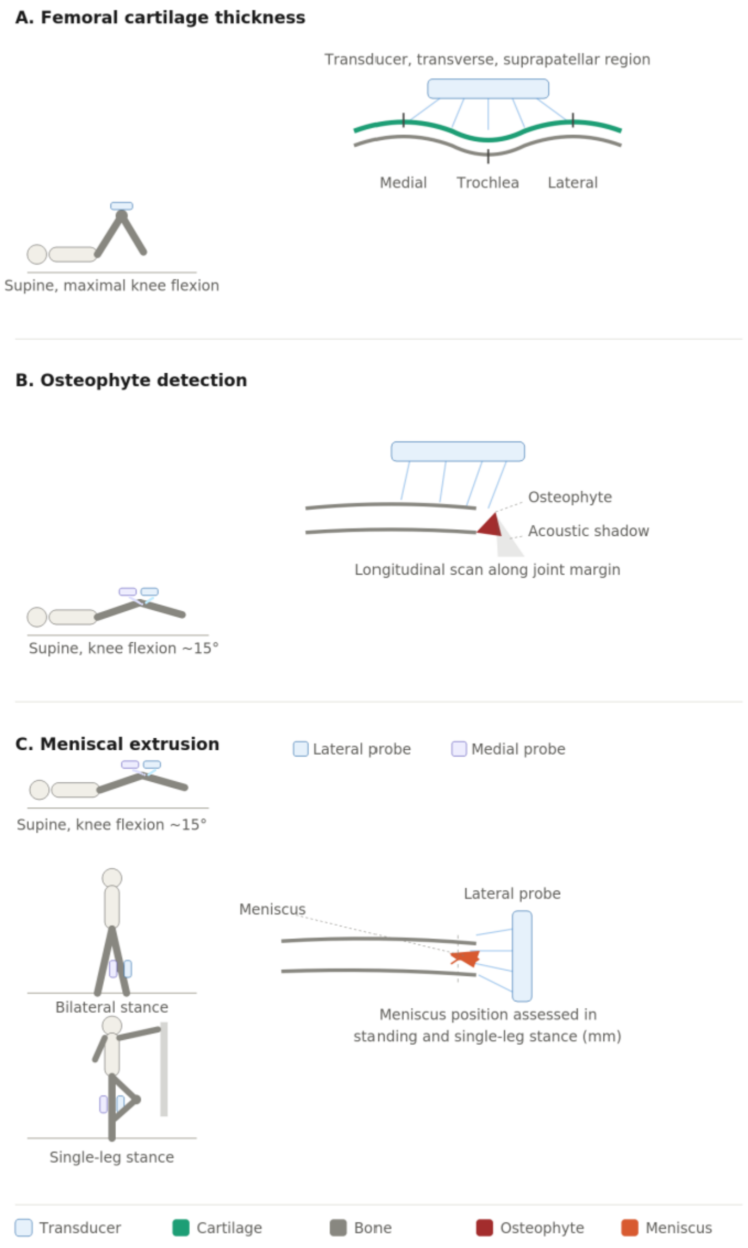

(A) Femoral cartilage thickness: supine, knee maximally flexed, transducer placed transversely over the suprapatellar region; thickness measured at the medial condyle, intercondylar (trochlear) region and lateral condyle. (B) Osteophyte detection: supine, knee flexed ~15°, longitudinal scan along the medial/lateral joint margin; osteophytes identified as bony step-ups, with or without posterior acoustic shadowing. (C) Meniscal extrusion: assessed supine, in bilateral standing, and in single-leg weight-bearing, with the transducer placed over both the lateral and medial compartments.
